# Supplementary material for: Massively parallel reporter assay for mapping gene-specific regulatory regions at single nucleotide resolution
Source: bioRxiv. 2025 May 14:2025.05.13.653746. Preprint. [Version 2] doi: 10.1101/2025.05.13.653746 (PMC12132421; doi:10.1101/2025.05.13.653746)
Supplement: Supplement 1 — Supplemental Figure 2S. Grm6, Vsx2, and Cabp5 LS-MPRAs to identify CRMs in the neonatal mouse retina. Barcode enrichment plots from the Grm6 (A), Vsx2 (B), and Cabp5 (C) LS-MPRAs, aligned with genome browser tracks and annotated with (i) known regulatory regions, (ii) coverage of the barcode-fragment association library across the locus, (iii) log2 base conservation across 60 vertebrate or 40 placental mammal species, (iv) regions of open chromatin in the P8 mouse retina, and (v) RefSeq gene models. Expanded regions of interest (yellow dashed boxes) show peaks or regions that align with known regulatory elements for each gene. Supplemental Figure 5S. Activity of backbone plasmids containing the Olig2 minimal promoter and EGFP. (A) Representative transverse sections of E14 retinas incubated in vitro for 24 hours show sparse GFP RNA and GFP fluorescence driven by control plasmids containing either EGFP alone or EGFP under the Olig2 minimal promoter. Pie charts of the percentage of electroporated cells with detectable GFP expression. Supplemental Figure 8S. TF Binding sites in Olig2-NR1 and NR3 CRMs. (A, H) TF binding motifs identified within Olig2-NR1 (A) and Olig2-NR3 (H) CRM candidates, aligned with the average d-MPRA plot (from Fig. 7). (B-E, G, I-K) Position frequency matrices of transcription factors aligning to Olig2-NR1: Sox4/11 to Motifs 13 and 16 (B), Lhx2 and Dlx2 to Motif 11 (C), Isl1 to Motif 8 (D), Foxp1 to Motif 18 (E), Mybl1 to Motif 2 (G), and Otx2 to Motif 12 (G); or Olig2-NR3: Ngn2 to Motifs 3 and 5 (I), Bhlhe22 to Motif 13 (J), and Lhx9 to Motif 15 (K). (F, L) Co-expression (pink) of Olig2 (blue) with Sox11, Sox4, Lhx2, Dlx2, Isl1, or Foxp1 (red) for Olig2-NR1 (F) and Ngn2, Bhlhe22, or Lhx9 (red) for Olig2-NR3 (L) on UMAP visualization of E14 mouse retinal gene expression. Supplemental Figure 10S. Activity of OLIG2 CRMs in OLIG2+ cells within embryonic chick spinal cords. (A) Schematic of an E2 chick transverse spinal cord with the field of view (re [file NIHPP2025.05.13.653746v2-supplement-1.pdf]

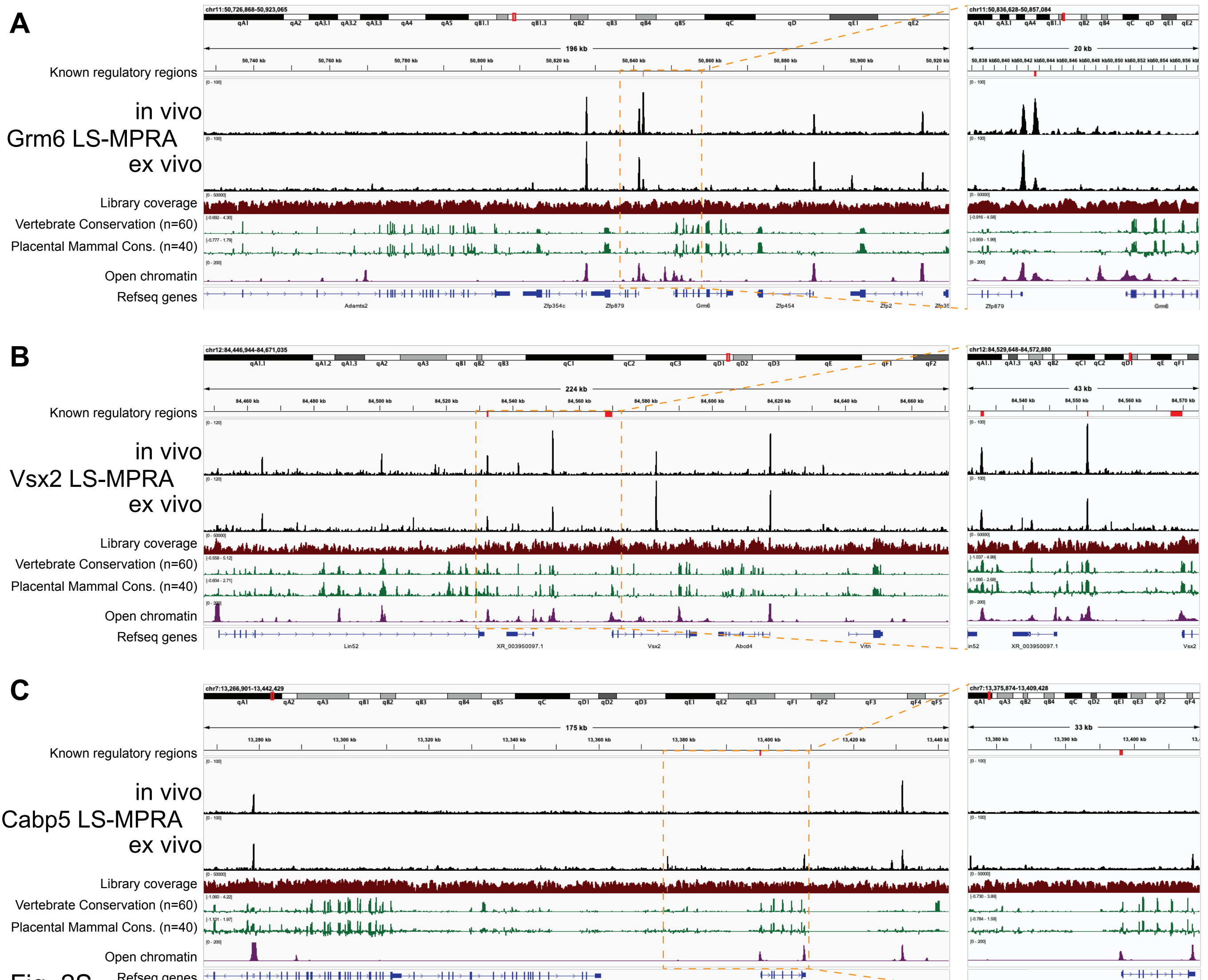
A
B
C
Known regulatory regions
in vivo
Grm6 LS-MPRA
ex vivo
Library coverage
Vertebrate Conservation (n=60)
Placental Mammal Cons. (n=40)
Open chromatin
Refseq genes
chr11:50,726,868-50,923,065
196 kb
chr11:50,836,628-50,857,084
20 kb
Adamts2
Zfp354c
Zfp879
Grm6
Zfp454
Zfp2
in vivo
Vsx2 LS-MPRA
ex vivo
chr12:84,446,944-84,671,035
224 kb
chr12:84,529,648-84,572,880
43 kb
Lin52
XR_003950097.1
Vsx2
Abcd4
Vrtn
in vivo
Cabp5 LS-MPRA
ex vivo
chr7:13,266,901-13,442,429
175 kb
chr7:13,375,874-13,409,428
33 kb
Zswim9
Lig1
Pla2g4c
Cabp5

Fig. 2S

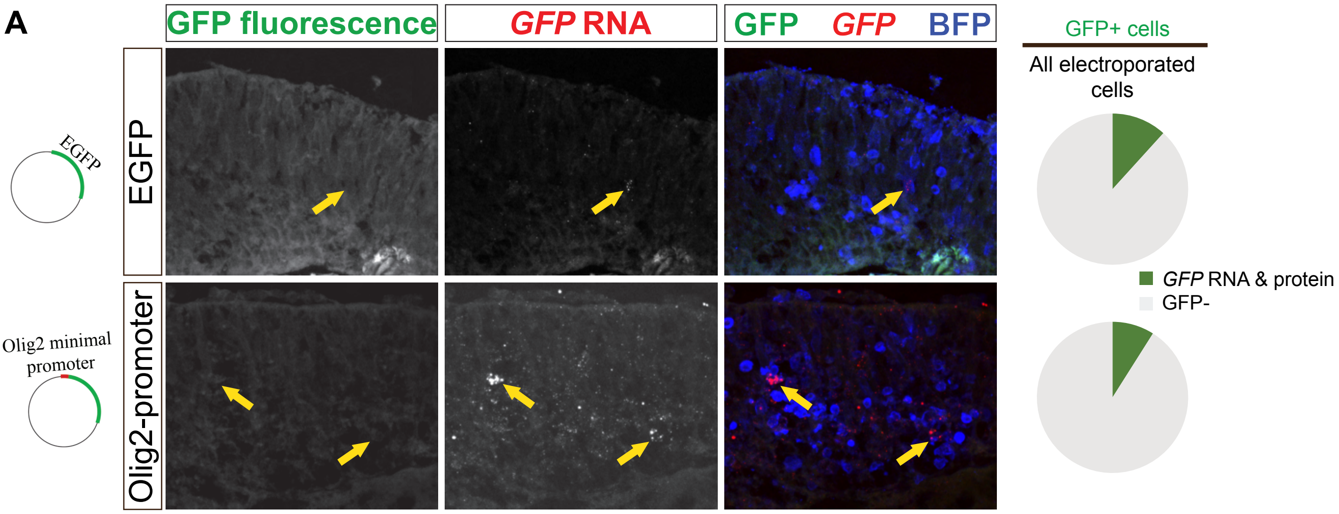
A
GFP fluorescence
GFP RNA
GFP GFP BFP
GFP+ cells
All electroporated cells
EGFP
EGFP
Olig2 minimal promoter
Olig2-promoter
GFP RNA & protein
GFP-

Fig. 5S

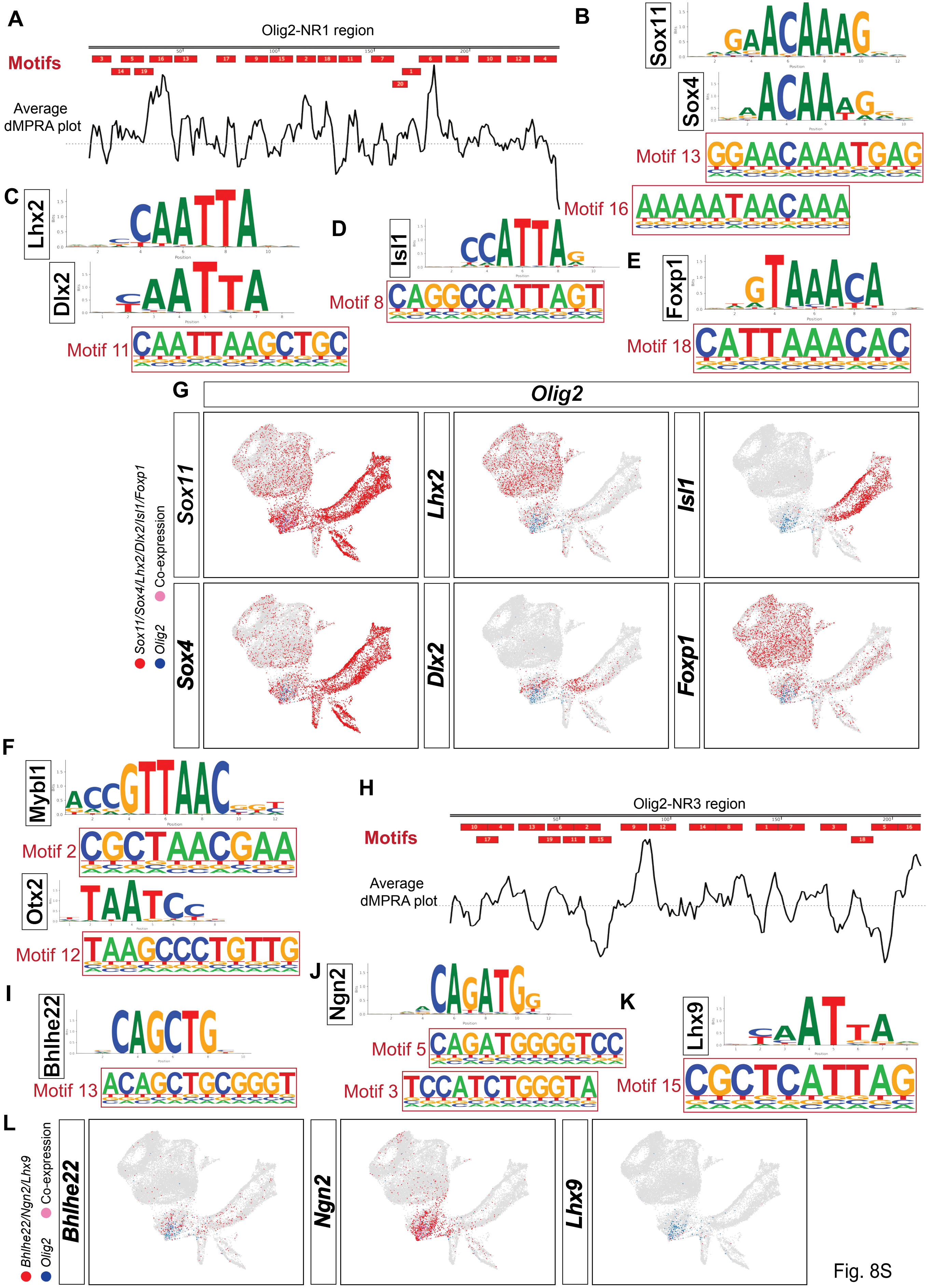

A
Olig2-NR1 region
Motifs
Average dMPRA plot
B
Sox11
Sox4
Motif 13
Motif 16
C
Lhx2
Dlx2
Motif 11
D
Isl1
Motif 8
E
Foxp1
Motif 18
G
Olig2
Sox11
Lhx2
Isl1
Sox4
Dlx2
Foxp1
Sox11/Sox4/Lhx2/Dlx2/Isl1/Foxp1
Olig2
Co-expression
F
Mybl1
Motif 2
Otx2
Motif 12
H
Olig2-NR3 region
Motifs
Average dMPRA plot
I
Bhlhe22
Motif 13
J
Ngn2
Motif 5
Motif 3
K
Lhx9
Motif 15
L
Bhlhe22/Ngn2/Lhx9
Olig2
Co-expression
Bhlhe22
Ngn2
Lhx9

Fig. 8S

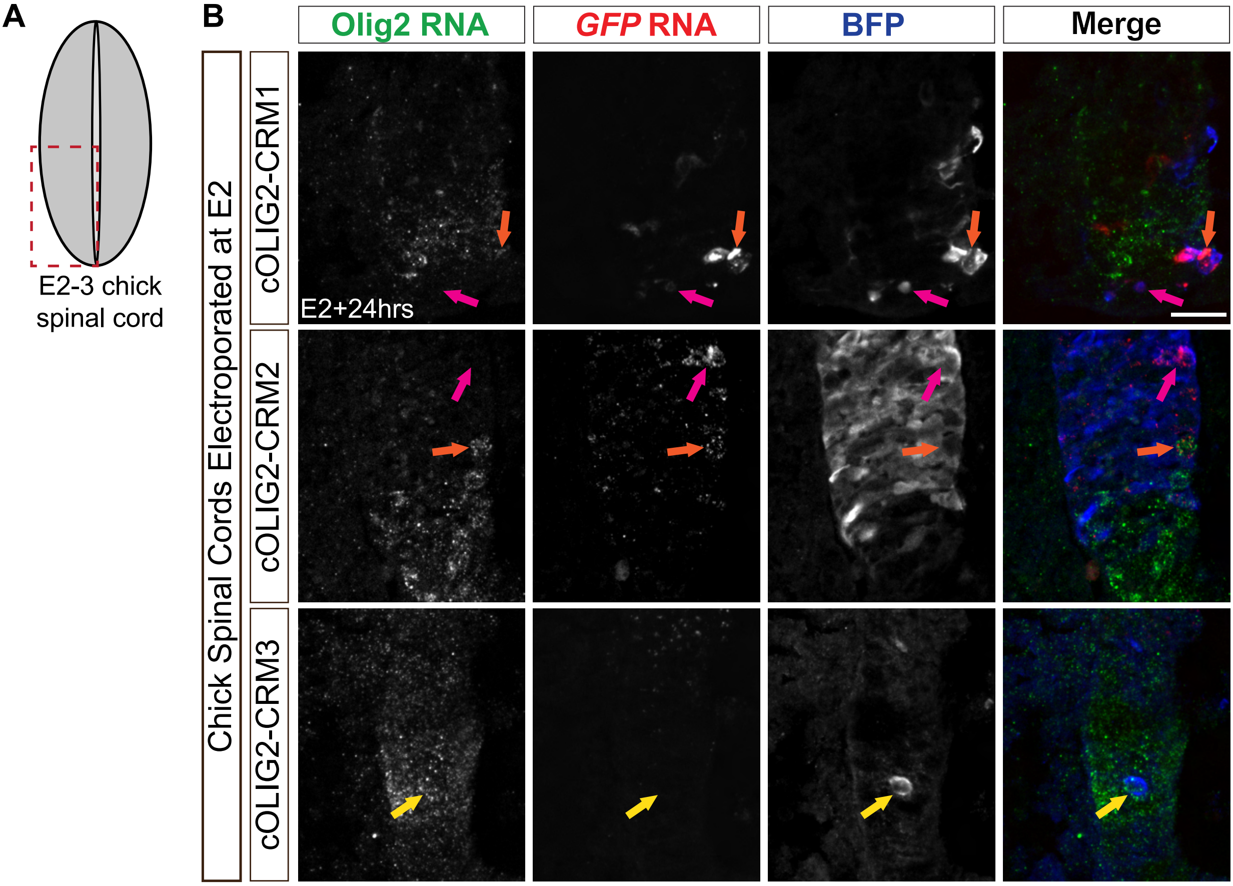
A
E2-3 chick spinal cord
B
Olig2 RNA
GFP RNA
BFP
Merge
Chick Spinal Cords Electroporated at E2
cOLIG2-CRM1
cOLIG2-CRM2
cOLIG2-CRM3
E2+24hrs

Fig. 10S

bioRxiv preprint doi: https://doi.org/10.1101/2025.05.13.653746; this version posted May 14, 2025. The copyright holder for this preprint (which was not certified by peer review) is the author/funder, who has granted bioRxiv a license to display the preprint in perpetuity. It is made available under aCC-BY 4.0 International license.

Table S1. Primer sequences

Method	Primer Name	Sequence
Stagintbc7 vector design	bc7_ins1	GGCGCGCCaGATCGGAAGAGCACACGTCTGAACTCCAGTCACCCTTACTTGTACAGCTCGTCCATG
	bc7_ins2	CCGATCtGGCGCGCCSWSWSWSWSWSWSWSWSWSWSWSWaGATCGGAAGAGCGTCGTGTAGGGAAAGAGTGTCCGGCCCCTTGAGCATCT
	bc7_ins1A	GCCTGTATGGCGCGCCaGATCGGAAGAGCACACGTCTGAACTCCAGTCACCCTTACTTGTACAGCTCGTCCATG
	bc7_ins1B	CTTGCACAGGCGCGCCaGATCGGAAGAGCACACGTCTGAACTCCAGTCACCCTTACTTGTACAGCTCGTCCATG
	bc7_ins1C	TCTTCGTGGGCGCGCCaGATCGGAAGAGCACACGTCTGAACTCCAGTCACCCTTACTTGTACAGCTCGTCCATG
	bc7_ins1D	AGGTTCACGGCGCGCCaGATCGGAAGAGCACACGTCTGAACTCCAGTCACCCTTACTTGTACAGCTCGTCCATG
	bc7_ins2A	CCGATCtGGCGCGCCATACAGGCVHBDVHBDVHBDVHBDVHBDVHBDaGATCGGAAGAGCGTCGTGTAGGGAAAGAGTGTCCGGCCCCTTGAGCATCT
	bc7_ins2B	CCGATCtGGCGCGCCTGTGCAAGVHBDVHBDVHBDVHBDVHBDVHBDaGATCGGAAGAGCGTCGTGTAGGGAAAGAGTGTCCGGCCCCTTGAGCATCT
	bc7_ins2C	CCGATCtGGCGCGCCCACGAAGAVHBDVHBDVHBDVHBDVHBDVHBDaGATCGGAAGAGCGTCGTGTAGGGAAAGAGTGTCCGGCCCCTTGAGCATCT
	bc7_ins2D	CCGATCtGGCGCGCCGTGAACCTVHBDVHBDVHBDVHBDVHBDVHBDaGATCGGAAGAGCGTCGTGTAGGGAAAGAGTGTCCGGCCCCTTGAGCATCT
LS-MPRA Adapters	enh7_5'1S	CGATAGTCGACCAATTGCTCGAGGGTGACTGGAGTTCAGACGTGTGCTCTTCCGATC*t
	enh7_5'2phos	/5Phos/GATCGGAAGAGCACACGTCTGAACTCCAGTCACCCTCGAGCAATTGGTCGACTATCG
	enh7_3'1phos	/5Phos/GGCGCGCCattaaGCTGGTAGACTgcgatCT
	enh7_3'2S	AGatcgcAGTCTACCAGCttaatGGCGCGCC*t
Amplify fragments	enh7_ampF	CGATAGTCGACCAATTGCTC
	enh7_ampR	AGatcgcAGTCTACCAGC
d-MPRA assembly	Olig2_NR1_mutF	CGATAGTCGACCAATTGCTCGAcagacatcaaatcagtaagccctgttg
	Olig2_NR1_mutR	atcgcAGTCTACCAGCttaatTTGTTAGTGGCGTCTGTCCCG
	Olig2_NR2_mutF	CGATAGTCGACCAATTGCTCGAgagtggtcctcacatgcccaa
	Olig2_NR2_mutR	atcgcAGTCTACCAGCttaatCGTGTTTCCCAGCTGGCT
	Olig2_NR3_mutF	CGATAGTCGACCAATTGCTCGActaggtgcttggaccccatct
	Olig2_NR3_mutR	atcgcAGTCTACCAGCttaatAGTTGAGGCTTGGGATTCCGG
	MutF	CGATAGTCGACCAATTGCTCGA
	MutR	atcgcAGTCTACCAGCttaat
	StatadualA_gfpF	GCTGGTAGACTgcgatAcctcaCTATATAATGGAAGCTTG
	StatadualA_gfpR	CCTCGAGCAATTGGTCGACTATCGATTACTTGTACAGCTCGTCCATGCCG
LS-MPRA BC sequencing	bc7_cdnaR	AAGTCAGATGCTCAAGGGGC
	bc7_cdnaF	GCAGAAGAACGGCATCAAGG
	bc7_cdnaR2	GCCGGACACTCTTTCCCTAC
d-MPRA BC sequencing	bc7_cdnaR_WPRE	AGCAGCGTATCCACATAGCG
	bc7_cdnaF*	GCAGAAGAACGGCATCAAGG
	MutR*	atcgcAGTCTACCAGCttaat
	dualmut_IllR1F	ACACTCTTTCCCTACACGACGCTCTTCCGATCtACCAATTGCTCGAGG

bioRxiv preprint doi: https://doi.org/10.1101/2025.05.13.653746; this version posted May 14, 2025. The copyright holder for this preprint (which was not certified by peer review) is the author/funder, who has granted bioRxiv a license to display the preprint in perpetuity. It is made available under aCC-BY 4.0 International license.

	dualmut_IllR2F	ACACTCTTTCCCTACACGACGCTCTTCCGATCTACCAATTGCTCG ACA
	dualmut_IllR2R	GTGACTGGAGTTCAGACGTGTGCTCTTCCGATCtgcAGTCTACCAG Cttaat
Construct vector	compvec_F	CAGCTCACTCAAAGGCGGTA
	compvec_R	CCTCGAGCAATTGGTCGACTATC
Olig2-NR1 and -NR2 constructs	Olig2_NR1F	GACCAATTGCTCGAGGcagacatcaaatcagtaagccctgttg
	Olig2_NR1R	ATGTGCTTTTttgttagtggcgtctgtcccg
	Olig2_NR2F	GACCAATTGCTCGAGGgagtggtcctcacatgccc
	Olig2_NR2R	GGAACAATGTGCTTTTcgtgtttcccagctggc
	Olig2_minpr-GFPint	AAAAGCACATTGTTCCCGCC
	GFPint_vecR	GCCTTTGAGTGAGCTGgccctcccatatgtccttcc
Olig2-NR3 construct	Olig2_NR3F	GACATATGGGAGGGCCTAGGTGCTTGGACCCCATC
	Olig2_NR3R	GCCTTTGAGTGAGCTGagttgaggcttgggattccg
	Vec_GFPintF	gaccaattgctcgaggAAAAGCACATTGTTCCCGCC
	GFPint_R	GCCCTCCCATATGTCCTTCC
Olig2-composite construct	Olig2_NR1F*	GACCAATTGCTCGAGGcagacatcaaatcagtaagccctgttg
	Olig2comp_NR1R	GGACCACTCttgttagtggcgtctgtccc
	Olig2comp_NR2F	cactaacaaGAGTGGTCCTCACATGCCCa
	Olig2_NR2R*	GGAACAATGTGCTTTTcgtgtttcccagctggc
	Olig2_minpr-GFPint*	AAAAGCACATTGTTCCCGCC
	GFPint_R*	GCCCTCCCATATGTCCTTCC
	Olig2_NR3F*	GACATATGGGAGGGCCTAGGTGCTTGGACCCCATC
	Olig2_NR3R*	GCCTTTGAGTGAGCTGagttgaggcttgggattccg
Backbone control constructs	Vec_GFPintF*	gaccaattgctcgaggAAAAGCACATTGTTCCCGCC
	Vec_nominprGFPF	accaattgctcgaggGCCACCATGGTGAGCAAG
	GFPint_R*	GCCCTCCCATATGTCCTTCC
Ngn2-CRM1, -CRM2, and -CRM3 constructs	Ngn2-CRM1_F	ACCAATTGCTCGAGGgaagattgggagtatgaatagttgtc
	Ngn2-CRM1_R	atcgcAGTCTACCAGCaatgctcacatcctccctcag
	Ngn2-CRM2_F	ACCAATTGCTCGAGGtctcatcagtcccaaaactggc
	Ngn2-CRM2_R	atcgcAGTCTACCAGCaatgatggtgggatggagag
	Ngn2-CRM3_F	ACCAATTGCTCGAGGactcgggcccctaatgagc
	Ngn2-CRM3_R	atcgcAGTCTACCAGCtttatctaatcagcataaaatggttctaaagctcc
	TATAminpr_F	GCTGGTAGACTgcgatCT
	GFPint_vecR	GCCTTTGAGTGAGCTGgccctcccatatgtccttcc
Ngn2-CRM4 construct	Ngn2-CRM4_F	GACATATGGGAGGGCcctaaaagtgagaagcctggag
	Ngn2-CRM4_R	GCCTTTGAGTGAGCTGcaaggaggcaataaaccccc
	Vec_TATAminpr_F	GACCAATTGCTCGAGGGCTGGTAGACTgcgatCT
	GFPint_R*	GCCCTCCCATATGTCCTTCC
cOLIG2-CRM1 construct	cOLIG2_CRM1F	ACCAATTGCTCGAGGaccaaagagttaatttgcgtttttaaacc
	cOLIG2_CRM1R	CTTTATAGCCGCGCCGGGctgctgtaatgtttttgttatttttcctc
	cOLIG2_promF	cccggcgcggctataaaggc
	cOLIG2_promR	CTCACCATGGTGGCggctctcaccgtgctcgggg
	GFPint_F	GCCACCATGGTGAGCAAG
	GFPint_vecR*	GCCTTTGAGTGAGCTGGCCCTCCCATATGTCCTTCC
cOLIG2-CRM2 construct	cOLIG2_CRM2F	ACCAATTGCTCGAGGgttacgcgttacgatggatctgac
	cOLIG2_promR*	CTCACCATGGTGGCggctctcaccgtgctcgggg
	GFPint_F*	GCCACCATGGTGAGCAAG
	GFPint_vecR*	GCCTTTGAGTGAGCTGGCCCTCCCATATGTCCTTCC
cOLIG2-CRM3 construct	cOLIG2_CRM3F	GACATATGGGAGGGCgctcccattggtcagcgctg
	cOLIG2_CRM3R	GCCTTTGAGTGAGCTGagcccgtaatcccgcgc
	cOLIG2-vec_promF	ACCAATTGCTCGAGGcccggcgcggctataaaggc

bioRxiv preprint doi: https://doi.org/10.1101/2025.05.13.653746; this version posted May 14, 2025. The copyright holder for this preprint (which was not certified by peer review) is the author/funder, who has granted bioRxiv a license to display the preprint in perpetuity. It is made available under aCC-BY 4.0 International license.

	cOLIG2_promR*	CTCACCATGGTGGCggctctcaccgtgctcgggg
	GFPint_R*	GCCCTCCCATATGTCCTTCC
ddPCR	Olig2_qF	GCGAGCACCTCAAATCTAATTC
	Olig2_qR	AAAAGATCATCGGGTTCTGGG
	Hprt_qF	CCCCAAAATGGTTAAGGTTGC
	Hprt_qR	AACAAAGTCTGGCCTGTATCC
	*reused	

bioRxiv preprint doi: https://doi.org/10.1101/2025.05.13.653746; this version posted May 14, 2025. The copyright holder for this preprint (which was not certified by peer review) is the author/funder, who has granted bioRxiv a license to display the preprint in perpetuity. It is made available under aCC-BY 4.0 International license.

Table S2: Genome Coordinates for ROIs

Genome	Chromosome	Start	End	Description
mm10	chr6	115930332	115930431	Rho PPR (Zack et al., 1991)
	chr6	115931731	115931977	Rho RER (Nie et al., 1996)
	chr6	115928742	115929214	Rho CBR (Corbo et al., 2010)
	chr11	50842558	50842758	Grm6 CRM (Kim et al., 2008)
	chr12	84567682	84569914	Vsx2 2.4 kb promoter CRM (Rowan et al., 2005)
	chr12	84552103	84552267	Vsx2 164 bp enhancer CRM (Kim et al., 2008)
	chr12	84532102	84532711	Vsx2 ECR4 homologue (Buenaventura et al., 2018)
	chr12	84532049	84533013	Vsx2 CRC-SE-mR0-37 (Honnell et al., 2022)
	chr12	84550173	84553991	Vsx2 CRC-SE-mR3-17 (Honnell et al., 2022)
	chr7	13397865	13398310	Cabp5 CRM (Kim et al., 2008)
	chr16	91140523	91140769	Olig2-NR1
	chr16	91223668	91223849	Olig2-NR2
	chr16	91232270	91232482	Olig2-NR3
	chr16	91225474	91225600	Olig2 minimal promoter
	chr16	91235256	91238546	Olig2-K23 (Sun et al., 2006)
	chr16	91139504	91141503	Olig2-ULTRA (Chen et al., 2008)
	chr16	91223550	91225549	Olig2-basal promoter (Chen et al., 2008)
	chr16	91191767	91193766	Olig2-EC2 (Fan et al., 2023)
	chr16	91208209	91211318	Olig2-5F7 (Friedli et al., 2010)
	chr3	127628497	127632832	Ngn2-TgN2 (Simmons et al., 2001)
	chr3	127642407	127642608	Ngn2-CRM1
	chr3	127605638	127605854	Ngn2-CRM2
	chr3	127632596	127632883	Ngn2-CRM3
	chr3	127572578	127572810	Ngn2-CRM4
galGal6	chr1	106522687	106522790	cOLIG2 minimal promoter
	chr1	106486012	106486309	cOLIG2-CRM1
	chr1	106522473	106522687	cOLIG2-CRM2
	chr1	106531687	106531776	cOLIG2-CRM3
